# Optimizing physician-patient communication through an integrated perspective of prospect theory and the ultimatum game: a controlled study among patients undergoing elective surgery or invasive procedures in a Chinese tertiary hospital

**DOI:** 10.3389/fpubh.2026.1937493

**Published:** 2026-10-22

**Authors:** Muqing Niu, Panpan Liu

**Affiliations:** 1 The First Affiliated Hospital, and College of Clinical Medicine of Henan University of Science and Technology, Luoyang, China; 2 Business School, Henan University of Science and Technology, Luoyang, Henan, China

**Keywords:** physician-patient communication, prospect theory, risk communication, shared decision-making, trust in physicians, ultimatum game

## Abstract

This study developed a structured preoperative communication intervention based on the complementary mechanisms of prospect theory and the ultimatum game. It evaluated effects on risk understanding, trust in physicians, treatment decisions, and related clinical outcomes. A two-campus, campus-level randomized, parallel-group controlled design included 320 patients undergoing elective surgery or invasive procedures at a tertiary hospital in Henan Province, China. One campus was assigned to the intervention condition and the other to the control condition, with 160 patients enrolled at each campus. The primary outcomes were risk understanding and trust in physicians. Secondary outcomes were treatment acceptance, 30-day complications after surgery or invasive procedures, and medical disputes within 3 months. Between-group associations were assessed using multivariable linear regression, logistic regression, and a modified Poisson model. In patient-level adjusted analyses, risk understanding was higher in the intervention group (beta = 2.001, 95% CI: 1.869–2.133). Trust in physicians was also higher (beta =2.703, 95% CI: 2.579–2.828). Treatment acceptance was more likely (OR = 3.491, *P* = 0.001), and the risk of 30-day complications after surgery or invasive procedures was lower (RR = 0.413, *P* < 0.001). Because medical disputes were uncommon, the corresponding association requires confirmation in larger samples. Structured preoperative communication based on cognitive calibration and interactional fairness was associated with better risk understanding, greater trust in physicians, and higher treatment acceptance. It was also associated with a lower risk of 30-day complications after surgery or invasive procedures.

## 1 Introduction

Healthcare is highly specialized, uncertain, and characterized by substantial information asymmetry. Physician-patient communication supports informed treatment choices and is fundamental to healthcare quality and patient safety. This is especially important before elective surgery and invasive procedures. Patients must understand expected benefits, potential complications, and the possible consequences of refusing or delaying treatment. One-way disclosure may not produce accurate risk perceptions or realistic expectations. It may also prevent patients from fully expressing their values and practical concerns. Improving the comprehensibility of risk information, strengthening patient participation, and creating a responsive decision process are therefore important healthcare management issues.

Previous studies associate patient-centered communication with better physician-patient relationships, with patient trust playing an important role (1). Patient-level evidence also shows that effective communication can reduce negative stereotypes of healthcare professionals through interpersonal and institutional trust (2). Patient participation may further strengthen trust in physicians (3). Communication skills, caring, health education, continuity of care, and shared decision-making are important and modifiable contributors to trust (4). Recent reviews of shared decision-making and trust, together with systematic reviews of physician communication styles, support this view (5, 6). These findings suggest that communication quality is not simply about providing more information. Patients must understand the information, participate in decisions, and feel that their concerns receive a response.

Effective risk communication is particularly important for patients undergoing elective surgery or invasive procedures. Insufficient explanation may lead patients to underestimate treatment uncertainty and develop unrealistic expectations. Excessive emphasis on severe adverse outcomes may instead amplify concern about low-probability risks. When actual outcomes differ markedly from prior expectations, normal medical uncertainty may be interpreted as inadequate disclosure. This may affect trust, treatment choices, and subsequent cooperation. Preoperative communication should therefore address statistical risk understanding, emotional responses, and individual values. Two-way feedback should also confirm whether patients truly understand the key information.

Appropriate patient participation is therefore an important component of high-quality physician-patient communication. Shared decision-making requires physicians to provide professional evidence while supporting patients in expressing values, life goals, and risk attitudes. Treatment plans are then developed through a process of choice, options, and decision (7). Early studies suggested that shared decision-making could improve treatment agreement, adherence, and patient outcomes in some settings (8). As the field developed, patient decision aids were shown to improve knowledge and participation while reducing decisional conflict (9). Other evidence suggests neutral to positive effects on healthcare quality and health outcomes, usually without substantial increases in cost or consultation time (10). Digital decision-support tools provide additional ways to facilitate patient participation (11). Embedding shared decision-making training in medical education can also improve learners' knowledge, attitudes, confidence, and skills (12). However, implementation remains constrained by health literacy, clinician competence, time pressure, and organizational processes (13). This gap is particularly evident in surgery. A 2025 study across 65 hospitals and 30,957 patients found inadequate shared decision-making in 36.5%−57.7% of surgical patients, depending on the instrument (14). Research has also linked the quality of surgical shared decision-making with patient-reported outcomes (15). A 2026 meta-analysis of nearly 20,000 real clinical consultations showed that improvement in routine practice remains limited (16). A recent surgical systematic review reported consistent gains in knowledge, participation, satisfaction, and decisional conflict. Evidence for complications and length of stay, however, remains inconsistent (17).

Patient participation alone cannot explain all problems in physician-patient communication. Patients may also process risk information with systematic bias. Prospect theory proposes that decisions under uncertainty do not depend solely on objective probabilities and final utility. They are influenced by reference dependence, loss aversion, and non-linear probability weighting (18). Cumulative prospect theory further explains how people subjectively weight probabilities and outcomes under uncertainty (19). In health communication, message framing has long explained different responses to equivalent information presented as gains or losses (20). Later studies showed that framing effects depend on message characteristics and specific health contexts (21). Perceived risk may also modify the effects of message framing (22). Recent research on risky-choice framing again demonstrates strong context dependence (23). Healthcare studies have increasingly incorporated framing and risk-perception mechanisms into interventions for real health decisions (24). More direct clinical evidence comes from surgical and interventional risk communication. A 2024 systematic review included 14 studies and 1,513 patients, including nine randomized trials. Seven randomized trials found that risk-communication aids improved understanding or recall of statistical risk (25). Prospect theory should therefore not be used simply to frame messages to persuade patients. It is better suited to establishing clear references, balancing benefits and risks, and calibrating judgments about low-probability events.

Treatment decisions also depend on whether patients feel that their concerns are respected and that the decision process is fair. The classic ultimatum game shows that people evaluate both the final payoff and the fairness of a proposal. If perceived unfairness exceeds an acceptable threshold, individuals may reject a proposal even when rejection causes personal loss (26). Experimental evidence further shows that fairness judgments influence behavioral and neural responses to unfair proposals (27). Clinical care is not an economic resource-allocation game, but the interaction structure has similarities. It can involve a physician proposal, patient feedback, physician explanation or adjustment, and a final decision. Qualitative research on breast cancer surgery found that decisions are shaped by information exchange, values clarification, decision support, and individual and system factors. Shared decision-making remains incompletely implemented in practice (28). A systematic review of breast reconstruction showed that shared decision-making reduced decisional conflict and regret while improving participation and knowledge (29). Prospect theory therefore explains mainly how patients understand risk. Fairness preferences and feedback in the ultimatum game help explain how patients evaluate and respond to the decision process. The two perspectives are theoretically complementary.

The effects of physician-patient communication may extend from risk perception and trust to treatment behavior and patient safety. Preoperative education has been associated with shorter mean hospital stays. Studies of abdominal surgery also reported lower odds of postoperative complications, while calling for stronger prospective evidence (30). A systematic review in abdominal surgery found improvements in length of stay, complications, anxiety, and psychological distress in some studies. However, intervention formats were heterogeneous and the overall evidence quality was limited (31). A meta-analysis of 37 randomized trials in orthopedic surgery found some improvement in early postoperative pain. Benefits for long-term pain and functional recovery were less consistent (32). A systematic review and meta-analysis in vascular surgery also found that preoperative education improved understanding of surgery-related knowledge (33). In the same year, a review of 40 studies and 4,113 patients combined conventional and network meta-analysis. Different forms of preoperative education may improve length of stay, analgesic use, pain, anxiety, satisfaction, and knowledge. Effects varied across educational approaches (34). These findings support a possible communication-cognition-behavior-clinical outcome pathway. They also justify caution when interpreting hard clinical endpoints such as complications.

Three related gaps remain in the literature on physician-patient communication. First, risk-communication research mainly explains how patients process probabilities, gains, and losses. Studies of shared decision-making and trust instead emphasize participation, responsiveness, and values. A unified framework that addresses both biased risk perception and imbalanced interaction is still lacking. Second, evidence on shared decision-making continues to grow. However, procedural fairness and the proposal-feedback-revision mechanism are rarely translated systematically into structured preoperative communication. Third, previous studies focus mainly on proximal outcomes such as knowledge, satisfaction, trust, or decisional conflict. Links across risk understanding, trust, treatment acceptance, patient safety, and medical disputes have not been evaluated systematically. Medical disputes are particularly important because they are infrequent downstream outcomes influenced by many clinical and organizational factors. Their relationship with communication interventions therefore requires separate testing and cautious interpretation.

To address these gaps, this study integrated prospect theory with the ultimatum game. We developed a dual-mechanism structured communication model combining cognitive debiasing with interactional fairness. The prospect theory module used dual reference points, balanced benefit-risk presentation, and calibration of low-probability risks to improve risk understanding. The ultimatum-game module used a two-way sequence of physician proposal, patient feedback, physician response or explanatory revision, and consensus. This process was designed to strengthen participation, responsiveness, and procedural fairness. The study included 320 patients undergoing elective surgery or invasive procedures at a tertiary hospital in Henan Province, China. The intervention and control groups each included 160 patients. Outcomes were risk understanding, trust in physicians, treatment acceptance, 30-day complications after surgery or invasive procedures, and medical disputes within 3 months. Multivariable regression, modified Poisson sensitivity analyses, and exploratory interaction analyses assessed robustness and heterogeneity.

This study makes three main contributions. First, it integrates prospect-theory explanations of biased risk perception with procedural fairness and interactive feedback from the ultimatum game. The framework therefore addresses two connected processes: how patients understand risk and how they evaluate and respond to treatment proposals. Second, the empirical analysis extends beyond proximal communication outcomes, including risk understanding and trust in physicians. It also examines treatment acceptance, 30-day complications after surgery or invasive procedures, and medical disputes as behavioral, clinical, and exploratory governance outcomes. Multivariable adjustment, sensitivity analyses, and heterogeneity tests improve the completeness and robustness of the evaluation. Third, the study reframes physician-patient communication as a service process rather than an exchange driven mainly by individual physician experience. This process can be standardized, evaluated, and embedded in systems for healthcare quality, patient safety, and risk governance. The findings provide empirical support for improving preoperative communication and patient participation in hospitals. Figure 1 presents the overall study framework.

**Figure 1:**
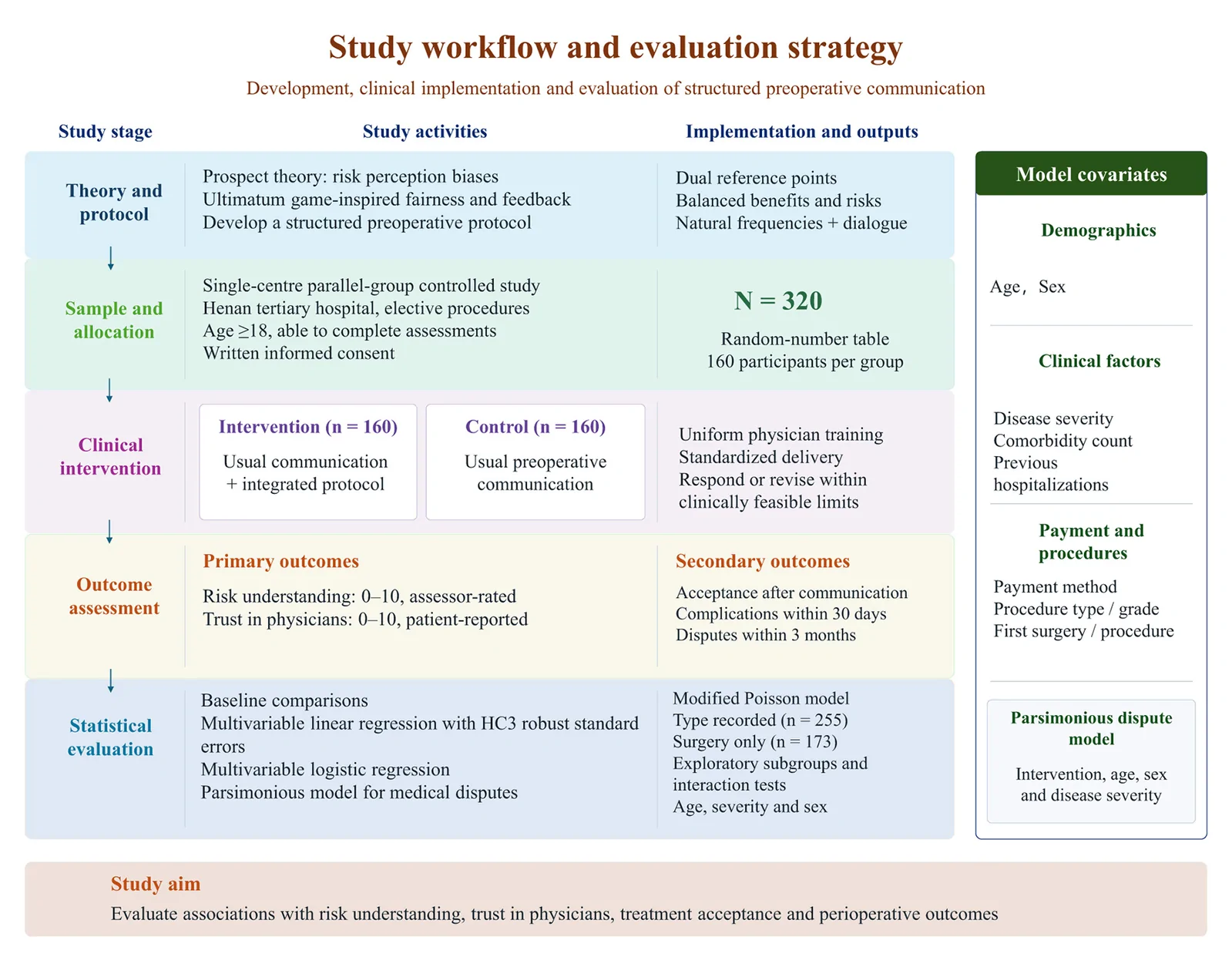
Study workflow and evaluation strategy.

## 2 Theoretical foundation and integration logic

### 2.1 Prospect theory: explaining patient risk perception and decision bias

Prospect theory was proposed by Kahneman and Tversky. It argues that people under uncertainty do not make decisions solely from the absolute utility of final outcomes. Instead, they evaluate possible outcomes relative to a subjective reference point and classify them as gains or losses. This process produces asymmetric value judgments. Compared with expected utility theory, prospect theory emphasizes psychological features observed in real decisions. These include reference dependence, loss aversion, diminishing sensitivity, and non-linear probability weighting (18, 19). It can therefore explain systematic cognitive biases when patients evaluate surgical benefits, treatment risks, and uncertain clinical outcomes. The prospect-theory value function is commonly expressed as follows:


$$\begin{array}{l} u\left(x\right)\;=\;\{\begin{array}{c} x^{\alpha },\;x\geq 0\\ -\lambda {\left(-x\right)}^{\beta },x<0 \end{array} \end{array}$$

Here, *x* represents the change in a treatment outcome relative to the patient's subjective reference point. When *x*≥0, the outcome is perceived as a gain. When *x* < 0, it is perceived as a loss. α and β represent value sensitivity in the gain and loss domains, respectively. They usually satisfy 0 < α and β < 1. λ is the loss-aversion coefficient and usually satisfies λ > 1. This indicates that an equivalent loss has a greater psychological effect than an equivalent gain. In this clinical context, *x* does not represent monetary gain. It represents the patient's subjective evaluation of treatment benefit, postoperative functional improvement, complications, and consequences of no treatment.

The value function has three main features. (1) Reference dependence: judgments of surgical gains and losses depend on a subjective reference point rather than objective outcomes alone. Examples include recovery times among similar patients or preoperative quality-of-life baselines. (2) Loss aversion: people generally assign greater subjective weight to a potential loss than to an equivalent potential gain. (3) Diminishing sensitivity: patients may be more psychologically sensitive to low-probability but severe risks than to moderate-probability but minor risks.

Beyond the value function, prospect theory proposes that subjective perceptions of objective probabilities are non-linear. A commonly used probability-weighting function is expressed as follows:

$$\begin{array}{l} w\left(p\right)\;=\;\frac{p^{\gamma }}{{\left[p^{\gamma }+{\left(1-p\right)}^{\gamma }\right]}^{\frac{1}{\gamma }}} \end{array}$$

Here, *p* is the objective probability of a clinical event. *w*(*p*) is the decision weight after subjective cognitive processing. γ is the probability-weighting parameter and captures the non-linear transformation from objective probability to subjective decision weight. When *w*(*p*) ≠ *p*, the patient's psychological perception of probability does not fully correspond to the objective probability.

This phenomenon is particularly important in medical decisions. Patients may overweight low-probability events with severe consequences. For example, they may focus on a 1% risk of a serious complication while overlooking a higher risk of disease progression without treatment. Patients may also underestimate adverse outcomes when physicians overemphasize treatment success rates. A marked gap between actual outcomes and prior expectations can then create perceptions of inadequate risk disclosure or broken promises.

This study therefore does not use prospect theory simply to persuade patients through gain or loss framing. Framing effects depend on risk level, context, and information presentation. No single frame has a stable advantage across all medical settings (20–23). We instead use clear references, balanced presentation of risks and benefits, and accessible probability expressions to calibrate risk perception. This approach is consistent with recent research emphasizing better understanding of statistical risk before surgery and interventional procedures (25).

### 2.2 The ultimatum game: procedural fairness and physician-patient interaction

The ultimatum game is mainly used to explain concerns about fairness, participation, and responsiveness. In the classic game, a responder may reject a proposal that provides a material benefit if it is perceived as clearly unfair (26). Decisions therefore depend on both final outcomes and the fairness of the process (27). Clinical care is not equivalent to an economic resource-allocation game, but the interaction structure is similar. It can be described as proposal, response, revision, and acceptance or rejection. In clinical settings, the first step is the physician's initial proposal. The physician explains treatment benefits, potential risks, and alternatives based on professional knowledge. The second step is the patient's response. Patients express questions and preferences based on risk attitudes, occupational needs, financial circumstances, family responsibilities, and other factors. The third step is physician explanation or adjustment. The physician responds to concerns and supplements or revises the plan within clinically feasible limits. The fourth step is the final decision. Patients make a choice after adequate understanding and after feeling that their views have received a response.

In this study, the utility of a patient's treatment decision is represented as follows:

$$\begin{array}{l} U_{patient}\left(S\right)\;=\;B_{w}\left(S\right)-R_{w}\left(S\right)-\theta F\left(S\right) \end{array}$$

Here, *S* denotes the treatment plan proposed by the physician. *B*_*w*_(*S*) denotes the patient's cognitively processed evaluation of the expected treatment benefit. *R*_*w*_(*S*) denotes the patient's subjective evaluation of treatment risks and potential losses. *F*(*S*) denotes the perceived degree of procedural unfairness. θ denotes the patient's sensitivity to unfairness.

Patients are more likely to accept a treatment plan when they perceive greater benefits, acceptable risks, and adequate responses to their views. In this study, the ultimatum game therefore corresponds to patient participation, two-way feedback, procedural fairness, and trust in physicians. Trust in physicians is the main proximal outcome of this mechanism and may subsequently influence treatment acceptance.

### 2.3 Integrating prospect theory and the ultimatum game

The central problem in traditional physician-patient communication arises from the combined effects of information asymmetry and cognitive bias. Integrating prospect theory with the ultimatum game provides complementary explanations and intervention mechanisms. These mechanisms operate at two main levels.

The first pathway calibrates cognitive bias and is grounded in prospect theory. It establishes clear risk references and presents treatment benefits and potential losses in a balanced manner. It also uses more understandable probability formats, such as natural frequencies, to reduce bias from a single frame (18, 19, 25).

The second pathway focuses on interactional fairness. It draws on fairness preferences and response mechanisms in the ultimatum game. It also incorporates patient participation, values clarification, and two-way information exchange from shared decision-making. The resulting process follows proposal, patient feedback, physician response or explanatory revision, and final decision (7, 26–29).

The integrated framework aims to improve both the quality of patient risk perception and the level of interactional trust. It is also intended to improve the treatment decision process. Figure 2 presents the integrated theoretical framework.

**Figure 2:**
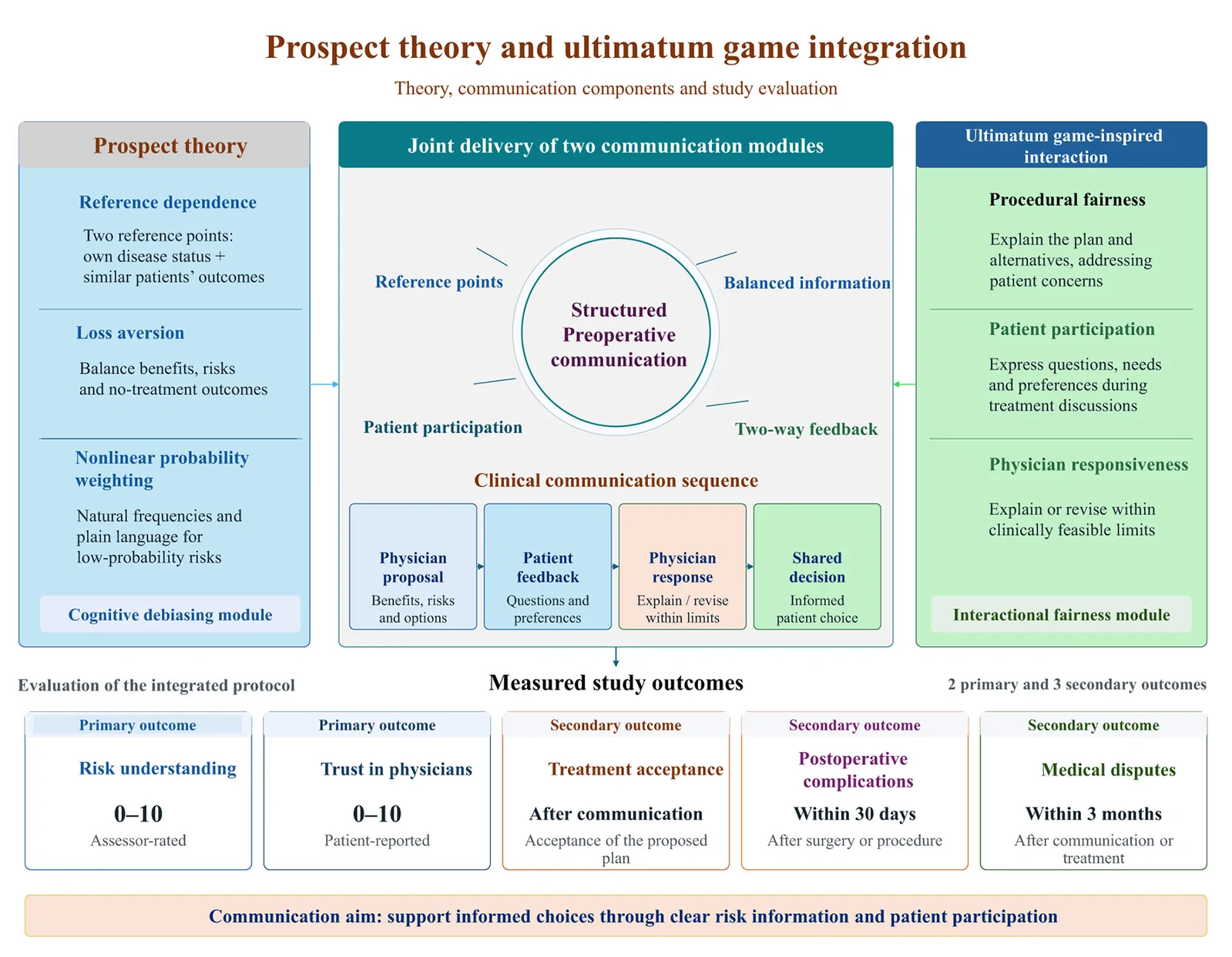
Integrated theoretical framework.

## 3 Study design

### 3.1 Study design and participants

This cluster-randomized, parallel-group controlled study was conducted across two campuses of the same tertiary hospital in Henan Province, China. The two geographically separate hospital campuses participated in the study. Before patient enrollment, the campuses were randomized at the campus level using a random-number table. One campus was assigned to the intervention condition and implemented the structured physician-patient communication model based on prospect theory and the ultimatum game, whereas the other campus was assigned to the control condition and continued routine preoperative physician-patient communication. Patients entered the corresponding study group according to the campus at which they received care.

Eligible participants were patients scheduled to undergo elective surgery or an elective invasive diagnostic or therapeutic procedure. The inclusion criteria were as follows: (1) age ≥18 years. (2) scheduled for elective surgery or an invasive diagnostic or therapeutic procedure; (3) conscious and able to communicate and comprehend normally; (4) able to complete the relevant study assessments independently; and (5) willing to participate and able to provide written informed consent. The exclusion criteria were as follows: (1) emergency surgery or an emergency invasive procedure; (2) severe cognitive impairment or psychiatric disorders that prevented effective physician-patient communication; (3) severe hearing, language, or other impairments that prevented completion of study assessments; or (4) inability to complete the required follow-up. A total of 320 patients were included, with 160 patients enrolled at the intervention campus and 160 at the control campus, corresponding to 160 patients in each study group. Complete data were available for all primary and secondary outcomes.

### 3.2 Group allocation and intervention

The intervention and control conditions were implemented at two separate campuses of the same hospital and were delivered by different physician teams. The physicians involved at the two campuses were comparable in professional qualifications, clinical level, and professional rank. Because each campus implemented only one study condition, the physical and personnel separation between the two campuses reduced the likelihood of cross-contamination between the intervention and control conditions. At the control campus, physicians continued routine preoperative communication throughout the study period. They explained the patient's condition, the need for surgery or an invasive procedure, potential benefits, risks, and relevant precautions, after which standard informed-consent procedures were completed.

At the intervention campus, the structured communication protocol was implemented in addition to routine preoperative communication. The prospect-theory module established dual reference points using the patient's condition and clinical outcomes in similar patients. Treatment benefits, potential risks, and possible consequences of no treatment were presented together. Low-probability risks were explained using natural frequencies and accessible language. The ultimatum-game module followed four steps: physician proposal, patient questions and individual needs, physician response or explanatory revision, and shared decision-making. This structure was intended to facilitate patient participation in treatment discussions. Physicians at the intervention campus received standardized training in the structured communication protocol before study implementation to reduce variation in intervention delivery. The intervention was then delivered according to a standardized communication process.

### 3.3 Outcomes and covariates

Risk understanding and trust in physicians were the primary outcomes. Risk understanding was scored from 0 to 10. Higher scores indicated better understanding of treatment benefits, potential risks, and clinical uncertainty. Trust in physicians was also scored from 0 to 10, with higher scores indicating greater trust. Risk understanding was rated by an assessor using predefined criteria. Trust in physicians was self-reported by the patient.

Secondary outcomes included three measures. (1) Treatment acceptance indicated whether the patient ultimately accepted the proposed treatment after communication. (2) Thirty-day complications after surgery or invasive procedures were clinically recorded relevant complications within 30 days after surgery or an invasive procedure. (3) Medical disputes within 3 months were formal complaints or medical disputes occurring within 3 months after communication or treatment. Because medical disputes were infrequent and had complex causes, they were treated as an exploratory outcome. They were used mainly to examine directional differences and were not a core measure of intervention effectiveness. Covariates included age, sex, marital status, occupation, payment method, disease severity, type of surgery or invasive procedure, grade of surgery or invasive procedure, first-time surgery or invasive procedure status, number of comorbidities, and previous hospitalizations.

### 3.4 Sample size estimation

The original sample-size planning was based on a patient-level comparison of two groups for the continuous primary outcome. The formula was:

$$\begin{array}{l} n\;=\;\frac{2{\left(Z_{1-\frac{\alpha }{2}}+Z_{1-\beta }\right)}^{2}}{d^{2}} \end{array}$$

Here, *n* is the required sample size in each group. α is the type I error probability, 1−β is statistical power, and *d* is the expected standardized effect size. With a two-sided test of α = 0.05, power of 1−β = 0.80, and expected effect size of *d* = 0.50, about 63 participants were required per group. Allowing for sample loss, at least 64 participants were required in each group. The study actually included 160 participants per group and 320 in total. This exceeded the minimum required for the continuous primary outcomes. The calculation was based mainly on continuous outcomes and does not imply equal power for rare events such as medical disputes. Only 16 medical disputes occurred in this study, so this outcome was analyzed exploratorily. Because assignment was conducted at the campus level and only two campuses participated, this patient-level calculation did not incorporate an intracluster correlation coefficient and should not be interpreted as a formal cluster-randomized sample-size calculation.

### 3.5 Statistical analysis

Data were processed and analyzed using SPSS 26.0 and related statistical software. Continuous variables were summarized as mean ± standard deviation $\left(\bar{x}\;\pm \;s\right)$ or median and interquartile range, depending on distribution. Categorical variables were summarized as counts and percentages [*n*(%)]. Continuous baseline variables were compared using independent-samples *t* tests or Welch *t* tests. Skewed variables were compared using the Mann-Whitney U test. Categorical variables were compared using Pearson χ^2^, with Fisher's exact test when expected counts were small. All tests were two-sided, and *p* < 0.05 was considered statistically significant. Because study-condition assignment occurred at the campus level, the regression analyses were interpreted as patient-level adjusted comparisons between the two study conditions. With only two campuses and one campus assigned to each condition, campus-level effects could not be estimated separately from the study condition. The resulting estimates are therefore interpreted as adjusted between-group associations rather than definitive cluster-adjusted causal effects.

#### 3.5.1 Multivariable linear regression for continuous outcomes

Multivariable linear regression models were fitted for risk understanding and trust in physicians:

$$\begin{array}{l} Y_{i}\;=\;\beta_{0}+\beta_{1}T_{i}+\overset{K}{\underset{k=2}{\sum }}\beta_{k}X_{ki}+\varepsilon_{i} \end{array}$$

Here, *Y*_*i*_ denotes the risk-understanding or trust-in-physicians score for patient *i*. β_0_ is the intercept. *T*_*i*_ is the intervention indicator, coded 1 for the intervention group and 0 for the control group. *X*_*ki*_ denotes the kth covariate for patient *i*. β_*k*_ represents the conditional association between the corresponding covariate and the outcome. ε_*i*_ is the random error term.

With all other covariates held constant:

$$\begin{array}{l} \beta_{1}\;=\;E\left(Y|T=1,X\right)-E\left(Y|T=0,X\right) \end{array}$$

Thus, β_1_ is interpreted as the adjusted mean score difference between the intervention and control groups. For example, when β_1_ = 2.001 in the risk-understanding model, the intervention group scores about 2.00 points higher after adjustment. It should not be interpreted as a 200.1% increase. Scores ranged from 0 to 10 and could show heteroskedasticity. Some observations could also have relatively high leverage in this moderate-sized sample. We therefore used HC3 heteroskedasticity-consistent robust standard errors to calculate 95% CIs and *p* values. The HC3 correction changes standard errors, confidence intervals, and significance tests. It does not change ordinary least-squares point estimates of the regression coefficients.

#### 3.5.2 Logistic regression for binary outcomes

Multivariable logistic regression models were fitted for binary outcomes, including treatment acceptance, 30-day complications, and medical disputes:

$$\begin{array}{l} \ln\left[\frac{P\left(Y_{i}=1\right)}{1-P\left(Y_{i}=1\right)}\right]\;=\;\alpha_{0}+\alpha_{1}T_{i}+\overset{K}{\underset{k=2}{\sum }}\alpha_{k}X_{ki} \end{array}$$

Here, *P*_*i*_ = *P*(*Y*_*i*_ = 1) is the probability that patient *i* experiences the target outcome. $\frac{P_{i}}{1-P_{i}}$ is the odds of that outcome. *T*_*i*_ denotes intervention group, *X*_*ki*_ denotes the covariates, and α_1_ represents the log-odds association between the intervention and the outcome.

$$\begin{array}{l} \text{OR }=\;e^{\alpha_{1}} \end{array}$$

OR denotes the odds ratio. When OR > 1, the odds of the target event are higher in the intervention group. When OR < 1, the odds are lower. For example, an OR of 3.491 for treatment acceptance means that adjusted odds are about 3.49 times those in the control group. It does not mean that the probability of treatment acceptance is 3.49 times higher.

Adjusted between-group associations were reported as odds ratios (ORs) with 95% CIs. The main adjusted model included age, sex, disease severity, comorbidity count, previous hospitalizations, and payment method. It also included type of surgery or invasive procedure, grade of surgery or invasive procedure, and first-time surgery or invasive procedure status. Only 16 medical disputes occurred in the full sample. To reduce overfitting with sparse events, a parsimonious logistic model was used for this outcome. The model adjusted only for intervention, age, sex, and disease severity.

#### 3.5.3 Sensitivity analysis for postoperative complications

The 30-day complication rate after surgery or invasive procedures was 26.9% in the control group, so the outcome was not rare. Under this condition, the logistic OR may numerically overstate the relative effect compared with the adjusted risk ratio (RR). We therefore used modified Poisson regression with robust sandwich standard errors to estimate the adjusted RR directly. The model was:

$$\begin{array}{l} lnE\left(Y_{i}|X_{i}\right)\;=\;\gamma_{0}+\gamma_{1}T_{i}+\overset{K}{\underset{k=2}{\sum }}\gamma_{k}X_{ki} \end{array}$$

Here, *Y*_*i*_ indicates whether a 30-day complication after surgery or an invasive procedure occurred. *T*_*i*_ denotes intervention group, *X*_*ki*_ denotes the adjustment covariates, and γ_1_ is the intervention coefficient.

The adjusted risk ratio was:

$$\begin{array}{l} \text{RR }=\;e^{\gamma_{1}} \end{array}$$

When RR < 1, the risk of complications is lower in the intervention group. We also conducted two restricted-sample sensitivity analyses. The first included only patients with recorded type of surgery or invasive procedure. The second included only patients who clearly underwent surgery. We compared the direction, magnitude, and 95% CI of the RR across these sample definitions.

#### 3.5.4 Subgroup and interaction analyses

Exploratory subgroup analyses assessed whether between-group associations varied by age, disease severity, or sex. Age groups were < 45 years, 45–64 years, and >=65 years. Disease severity was classified as no severe/critical condition vs. severe/critical illness. Sex was classified as female or male. A significant result in one subgroup and a nonsignificant result in another does not establish a true difference between subgroups. We therefore formally tested interaction by adding study-condition-by-subgroup terms to the models:

$$\begin{array}{l} g\left[E\left(Y_{i}\right)\right]\;=\;\theta_{0}+\theta_{1}T_{i}+\theta_{2}S_{i}+\theta_{3}T_{i}S_{i}+\sum \theta_{k}X_{ki} \end{array}$$

Here, *S*_*i*_ denotes the subgroup variable and *T*_*i*_*S*_*i*_ is the interaction between intervention and subgroup characteristics. *g*(•) denotes the link function appropriate for the outcome. θ_3_ represents the difference in intervention effects across subgroups.

## 4 Results

### 4.1 Baseline characteristics

A total of 320 patients were included, with 160 in each group. No primary or secondary outcome data were missing. The groups were generally comparable in age, sex, marital status, disease severity, comorbidity count, and previous hospitalizations. Mean age was 56.81 ± 19.58 years in the intervention group and 55.63 ± 18.65 years in the control group (*P* = 0.583). Women accounted for 56.2% and 59.4% of the two groups, respectively (*P* = 0.651). Disease severity also did not differ significantly (*P* = 0.537). Patients without severe or critical illness accounted for 81.9% and 78.1% of the two groups. Severe illness accounted for 12.5% and 16.9%, and critical illness for 5.6% and 5.0%, respectively. Mean comorbidity counts were 3.29 ± 2.66 and 3.71 ± 2.87 (*P* = 0.170). Median previous hospitalizations were 0 [IQR 0–1] in both groups (*P* = 0.666). These results indicate only small differences in several key demographic and disease-burden measures. They provide a basic basis for subsequent comparisons of intervention effects. However, several baseline imbalances are relevant from a healthcare management perspective. Occupational composition differed significantly (*P* = 0.011). Retired patients were more common in the intervention group (33.8% vs. 18.8%), whereas farmers and unemployed patients were less common. This suggests heterogeneity in social roles and living circumstances. Occupational groups may differ in treatment decisions, time opportunity costs, and risk-communication needs. Payment method showed an even larger between-group difference (*P* < 0.001). Employee basic medical insurance covered 50.0% of the intervention group and 30.6% of the control group. Self-paying patients accounted for 1.9% and 10.6%, respectively. The healthcare payment environment was therefore not fully comparable between groups. Financial protection may accompany differences in treatment acceptance, risk sensitivity, and decision-making. Structured communication should therefore address potential differences in cost information, treatment choices, and benefit-risk trade-offs across payment groups. surgery or invasive procedure type also differed significantly (*P* < 0.001). Surgery was performed in 62.5% of the intervention group and 45.6% of the control group. Diagnostic procedures and unrecorded procedure types were more common in the control group. First surgery or invasive procedure status also differed (*P* < 0.001). A first surgery or invasive procedure was reported for 58.8% of the intervention group and 40.0% of the control group. Participants therefore differed in care pathways and previous healthcare experience. Procedure type and prior treatment experience may influence familiarity with risk information, communication needs, and treatment decisions. The overall difference in grade of surgery or invasive procedure approached statistical significance (*P* = 0.065). Grade III-IV surgery accounted for 57.5% of the intervention group and 50.0% of the control group. Clinical complexity may therefore not have been identical. Some type of surgery or invasive procedures and first-procedure status were unrecorded. This is both a missing-data issue and an indicator of room to improve structured clinical documentation. Evaluation of risk communication requires complete patient, care-process, and payment information as well as communication data. Subsequent multivariable models therefore adjusted for available demographic and clinical variables with baseline imbalance. This reduced potential influence from differences in patient composition and care pathways. The imbalances also support stratified patient management built on a common core communication process. A completely uniform communication approach may be less appropriate for routine hospital implementation (Table 1).

**Table 1:** Baseline characteristics of the intervention and control groups.

Characteristic	Intervention group (*n* = 160)	Control group (*n* = 160)	*P*-value
Age (years)	56.81 ± 19.58	55.63 ± 18.65	0.583
Number of comorbidities	3.29 ± 2.66	3.71 ± 2.87	0.170
Previous hospitalizations	0 [0–1]	0 [0–1]	0.666
Sex			0.651
Female	90 (56.2%)	95 (59.4%)	
Male	70 (43.8%)	65 (40.6%)	
Marital status			0.384
Married	133 (83.1%)	132 (82.5%)	
Unmarried	15 (9.4%)	8 (5.0%)	
Divorced	2 (1.2%)	3 (1.9%)	
Widowed	5 (3.1%)	8 (5.0%)	
Other	5 (3.1%)	9 (5.6%)	
Occupation			0.011
Farmer	38 (23.8%)	50 (31.2%)	
Retired	54 (33.8%)	30 (18.8%)	
Unemployed	16 (10.0%)	28 (17.5%)	
Student	10 (6.2%)	5 (3.1%)	
Employed/professional	28 (17.5%)	23 (14.4%)	
Self-employed/freelance	5 (3.1%)	9 (5.6%)	
Other	9 (5.6%)	15 (9.4%)	
Healthcare payment method			< 0.001
Resident basic medical insurance	77 (48.1%)	94 (58.8%)	
Employee basic medical insurance	80 (50.0%)	49 (30.6%)	
Self-pay	3 (1.9%)	17 (10.6%)	
Disease severity			0.537
No severe/critical condition	131 (81.9%)	125 (78.1%)	
Severe	20 (12.5%)	27 (16.9%)	
Critical	9 (5.6%)	8 (5.0%)	
Type of surgery or invasive procedure			< 0.001
Surgery	100 (62.5%)	73 (45.6%)	
Therapeutic procedure	19 (11.9%)	22 (13.8%)	
Diagnostic procedure	5 (3.1%)	20 (12.5%)	
Interventional treatment	12 (7.5%)	4 (2.5%)	
Unrecorded	24 (15.0%)	41 (25.6%)	
Grade of surgery or invasive procedure			0.065
Grade I–II	43 (26.9%)	38 (23.8%)	
Grade III–IV	92 (57.5%)	80 (50.0%)	
Ungraded/unrecorded	25 (15.6%)	42 (26.2%)	
First surgery or invasive procedure			< 0.001
Yes	94 (58.8%)	64 (40.0%)	
No	22 (13.8%)	20 (12.5%)	
Unrecorded	44 (27.5%)	76 (47.5%)	

Data are presented as mean ± standard deviation, median [IQR], or *n* (%). *P*-values represent overall between-group comparisons for each characteristic. The available patient-level data did not record education, household income, or physician-level variables.

### 4.2 Unadjusted outcome comparisons

In unadjusted comparisons, the intervention group performed better on both primary communication outcomes. The mean risk-understanding score was 7.87 ± 0.59 in the intervention group and 5.86 ± 0.50 in the control group. The mean difference (MD) was 2.01 points (95% CI: 1.89–2.13, *P* < 0.001), *Cohen*′*s d* = 3.66. Mean trust-in-physicians scores were 8.41 ± 0.58 and 5.69 ± 0.50, respectively. The MD was 2.72 points (95% CI: 2.61–2.84, *P* < 0.001), *Cohen*′*s d* = 5.03. These marked improvements show that preoperative communication quality should not be judged only by whether risk disclosure was completed. Evaluation should also consider whether patients understood the information and developed trust in physicians and the decision process. Physician-patient communication may therefore be transformed from experience-dependent interaction into a standardized and assessable healthcare service process.

For treatment decisions, 148/160 patients in the intervention group accepted the proposed treatment, for an acceptance rate of 92.5%. The corresponding rate was 78.8% in the control group. The absolute increase was 13.7 percentage points, and the unadjusted RR was 1.175 (95% CI: 1.072–1.288, *P* < 0.001). This difference suggests that clear risk explanation and opportunities for questions and participation may reduce hesitation or refusal. Such hesitation may arise from insufficient information, risk misjudgment, or inadequate trust. Earlier treatment decisions may also reduce time spent on repeated communication and reconsideration. This could improve coordination between preoperative preparation and subsequent care.

Thirty-day complications after surgery or invasive procedures occurred in 18 patients (11.3%) in the intervention group and 43 patients (26.9%) in the control group. The absolute risk reduction was 15.7 percentage points. The unadjusted RR was 0.419 (95% CI: 0.253–0.693, *P* < 0.001), corresponding to a relative risk reduction of about 58.1%. This finding extends the potential relevance of the communication intervention from patient experience to patient safety management. Better understanding of treatment risks and postoperative precautions may support preoperative preparation, postoperative activity, medication management, and recognition of abnormal symptoms. Preoperative communication may therefore represent a potential upstream component of perioperative safety management.

Medical disputes were uncommon. Within 3 months, six events (3.8%) occurred in the intervention group and ten (6.3%) in the control group. The absolute rate was about 2.4 percentage points lower in the intervention group. However, RR = 0.600 (95% CI: 0.223–1.612), and Fisher's exact test gave *P* = 0.443. The difference was not statistically significant. This result also shows the governance limits of structured communication. Better risk understanding and trust do not necessarily produce a parallel reduction in medical disputes. Disputes are also shaped by care quality, financial burden, expectations, complaint handling, and mediation mechanisms. Communication is therefore better positioned as an upstream preventive component of hospital conflict governance. It cannot replace organizational measures for quality control, complaint management, and dispute resolution (Table 2).

**Table 2:** Unadjusted outcome comparisons.

Outcome	Intervention group	Control group	Effect estimate (95% CI)	*P*
Risk-understanding score	7.87 ± 0.59	5.86 ± 0.50	MD 2.01 (1.89–2.13); d = 3.66	< 0.001
Trust-in-physicians score	8.41 ± 0.58	5.69 ± 0.50	MD 2.72 (2.61–2.84); d = 5.03	< 0.001
Treatment acceptance	148/160 (92.5%)	126/160 (78.8%)	RR 1.175 (1.072–1.288)	< 0.001
30-day complications after surgery or invasive procedures	18/160 (11.3%)	43/160 (26.9%)	RR 0.419 (0.253–0.693)	< 0.001
Medical disputes within 3 months	6/160 (3.8%)	10/160 (6.3%)	RR 0.600 (0.223–1.612)	0.443

MD, mean difference; d, Cohen's standardized mean difference; RR, risk ratio. Fisher's exact *P*-value is reported for medical disputes. Other categorical outcomes were tested using Pearson's chi-square test without continuity correction.

### 4.3 Adjusted between-group associations

After adjustment for measurable factors, associations between the integrated communication intervention and the primary outcomes remained stable. Covariates included age, sex, disease severity, comorbidity count, previous hospitalizations, payment method, and procedure-related factors. The adjusted coefficient was beta = 2.001 for risk understanding and beta = 2.703 for trust in physicians. Direction and magnitude changed little from the unadjusted estimates. The associations remained similar after adjustment for the measured patient- and procedure-level covariates; however, unmeasured campus-level differences could not be excluded. The structured intervention may have some process-level stability across different patient profiles. This supports converting risk communication from experience-dependent practice into a more standardized service process. For treatment acceptance, the adjusted OR was 3.491. The odds of accepting the proposed treatment remained substantially higher after adjustment for measurable confounders. Adequate explanation and patient participation may therefore improve more than subjective experience. They may also reduce decision hesitation caused by information asymmetry, risk misunderstanding, and insufficient trust. This may shorten repeated communication and reconsideration and improve coordination between preoperative decisions and subsequent care. The adjusted OR for 30-day complications was 0.335. Lower odds remained after adjustment for patient- and procedure-related factors. This finding extends the potential role of structured communication from patient-experience management to patient-safety management. Preoperative risk understanding, treatment preparation, and patient participation may be upstream components of perioperative safety. Hospitals could therefore monitor communication quality together with preoperative preparation, adherence, symptom recognition, and postoperative safety indicators. Satisfaction or informed-consent completion rates alone may be insufficient. For medical disputes, the parsimonious logistic model yielded an adjusted OR of 0.596. The point estimate remained below 1, but the confidence interval was wide and crossed 1. No independent statistical association was demonstrated. Improvements in risk understanding, trust, and treatment acceptance therefore do not automatically translate into fewer medical disputes. Medical disputes have more complex organizational and institutional determinants. These include care quality, financial burden, expectation management, complaint handling, and dispute mediation. Structured communication is therefore better viewed as an upstream management measure within hospital conflict governance. It cannot replace quality control, complaint management, or dispute-resolution mechanisms.

Overall, multivariable adjustment showed stable improvements in risk understanding, trust in physicians, and treatment acceptance. The intervention was also associated with fewer complications. However, its effect on medical disputes was not statistically supported. The model is therefore better positioned as a healthcare management tool for standardized communication, more efficient decisions, and potential patient-safety improvement. It should not be viewed solely as a method for preventing disputes (Table 3).

**Table 3:** Adjusted between-group associations after multivariable adjustment.

Outcome	Model	Adjusted association	95% CI	*P*-value
Risk-understanding score	Multivariable linear regression	β = 2.001	1.869–2.133	< 0.001
Trust-in-physicians score	Multivariable linear regression	β = 2.703	2.579–2.828	< 0.001
Treatment acceptance	Logistic regression	OR = 3.491	1.638–7.442	0.001
30-day complications	Logistic regression	OR = 0.335	0.181–0.620	< 0.001
Medical disputes	Parsimonious logistic regression	OR = 0.596	0.200–1.773	0.352

The main adjusted models included intervention, age per 10 years, sex, severe/critical illness, comorbidity count, previous hospitalizations, and employee medical insurance. They also included surgery vs. other/unrecorded procedures, Grade III–IV surgery or invasive procedure, ungraded/unrecorded surgery or invasive procedure, and first-time surgery or invasive procedure. A parsimonious model was used for medical disputes because events were sparse.

### 4.4 Sensitivity analysis of postoperative complications

The unadjusted analysis showed about a 58% lower risk of 30-day complications after surgery or invasive procedures in the intervention group. We therefore tested robustness using different models and restricted samples. The unadjusted RR in the full sample was 0.419, while multivariable logistic regression gave OR = 0.335. Because complications were not rare, the OR could overstate the relative effect. A modified Poisson model with robust sandwich standard errors was therefore used to estimate the RR directly. The adjusted RR was 0.413, which was very close to the unadjusted RR of 0.419. Relative-risk estimates were therefore stable after adjustment for available measurable covariates. In the first restricted analysis, 255 patients with recorded type of surgery or invasive procedure were included. The adjusted RR was 0.407. In the second, only 173 patients who clearly underwent surgery were included. The adjusted RR was 0.333. Effect directions were consistent across models and samples, and all 95% CIs excluded 1. The lower complication risk was therefore unlikely to be explained entirely by model choice, missing procedure type, or sample composition. Structured communication may have relevance beyond patient experience and may extend to perioperative safety management. Better understanding of risks, preoperative preparation, and postoperative precautions may strengthen patient participation and adherence. Physician-patient communication could therefore function as an upstream intervention in perioperative quality management. Hospitals implementing this model could jointly monitor communication quality, complications, treatment adherence, and recognition of abnormal symptoms. This would integrate communication more directly into patient-safety and quality-improvement systems. Nevertheless, these sensitivity analyses were conducted at the patient level and could not separate the study condition from unmeasured campus-level characteristics (Table 4).

**Table 4:** Sensitivity analyses of 30-day complications after surgery or invasive procedures.

Sensitivity analysis	*n*	Effect estimate	95% CI	*P*-value
Full sample, unadjusted	320	RR 0.419	0.253–0.693	< 0.001
Multivariable logistic model	320	OR 0.335	0.181–0.620	< 0.001
Modified Poisson with robust standard errors	320	RR 0.413	0.248–0.688	< 0.001
Patients with recorded procedure type only	255	RR 0.407	0.233–0.711	0.002
Surgical patients only	173	RR 0.333	0.168–0.661	0.002

Where estimable, restricted-sample modified Poisson models adjusted for intervention, age, sex, disease severity, comorbidity count, previous hospitalizations, and high procedure grade.

### 4.5 Exploratory subgroup and interaction analyses

Exploratory subgroup analyses showed generally consistent intervention directions across age, disease severity, and sex. Age showed a nominal interaction with risk understanding (*P* = 0.021). Improvement was relatively larger among patients aged 45–64 years. However, MDs were positive in all three age groups. The interaction therefore reflected differences in effect magnitude rather than a change in direction. Age interactions were not statistically significant for trust in physicians or 30-day complications. Among patients aged >=65 years, the 95% CI for the complication RR crossed 1. However, the overall age interaction was nonsignificant (*P* = 0.304). This result does not show that the intervention was ineffective in older patients. Interaction tests for disease severity and sex were also nonsignificant. The communication model may therefore have some applicability across patient groups. These findings support standardizing the core process while adapting how information is communicated. Hospitals can use common elements for benefit-risk explanation, patient feedback, and shared decision-making. Information format, explanation speed, and confirmation frequency can then be adapted to age, comprehension, and clinical complexity (Table 5).

**Table 5:** Exploratory subgroup effects and interaction tests.

Subgroup	*n* (intervention/ control)	Risk understanding: MD (95% CI)	Trust in physicians: MD (95% CI)	Complication RR (95% CI)
Age < 45 years	34/45	1.75 (1.53–1.97)	2.74 (2.46–3.02)	0.22 (0.05–0.92)
Age 45–64 years	59/51	2.21 (1.97–2.45)	2.87 (2.68–3.07)	0.32 (0.14–0.77)
Age >=65 years	67/64	2.00 (1.83–2.18)	2.58 (2.40–2.76)	0.64 (0.31–1.31)
No severe/critical condition	131/125	2.02 (1.89–2.16)	2.71 (2.58–2.84)	0.48 (0.27–0.84)
Severe/critical	29/35	1.99 (1.73–2.25)	2.80 (2.53–3.07)	0.28 (0.09–0.88)
Female	90/95	2.01 (1.86–2.17)	2.72 (2.57–2.87)	0.37 (0.18–0.74)
Male	70/65	2.01 (1.81–2.21)	2.74 (2.55–2.93)	0.49 (0.24–1.02)
Interaction *P*-value: age	-	0.021	0.097	0.304
Interaction *P*-value: disease severity	-	0.845	0.541	0.413
Interaction *P*-value: sex	-	0.971	0.860	0.567

## 5 Discussion

### 5.1 Main findings

This study developed a dual-mechanism structured communication model based on prospect theory and the ultimatum game. The model combined cognitive debiasing with interactional fairness. It was evaluated in 320 patients undergoing elective surgery or invasive procedures. The integrated intervention was significantly associated with better risk understanding, greater trust in physicians, and higher treatment acceptance. The intervention group also had a lower risk of 30-day complications after surgery or invasive procedures. This association remained stable in multivariable logistic regression, modified Poisson models, and restricted-sample sensitivity analyses. Medical disputes within 3 months were numerically less frequent in the intervention group. However, the adjusted difference was not statistically significant. Exploratory subgroup analyses showed generally consistent intervention directions across sex and disease-severity groups. Age showed only a nominal interaction with risk understanding. These findings suggest that structured communication may affect more than subjective experiences such as patient satisfaction. It may also influence risk understanding, physician-patient trust, treatment decisions, and potentially patient safety. However, these outcomes occupy different levels of evidence. Risk understanding and trust in physicians are proximal outcomes directly targeted by the intervention. Treatment acceptance is a behavioral outcome. Complications and medical disputes are more strongly influenced by additional clinical and organizational factors. Downstream outcomes therefore require increasingly cautious interpretation, especially reductions in complications or numerical declines in medical disputes. Because the intervention and control conditions were implemented at separate campuses, these findings should be interpreted as between-group associations within this two-campus study rather than as fully separable intervention effects.

### 5.2 Risk understanding and trust in physicians: from completing disclosure to managing communication quality

Risk understanding and trust in physicians were both markedly higher in the intervention group. Effect sizes changed little after adjustment for age, sex, disease severity, comorbidities, payment method, and procedure-related factors. This pattern is consistent with recent research on patient-centered communication and physician-patient trust. Previous studies show that patient trust does not depend simply on the amount of information provided. It is also related to opportunities for participation, physician responsiveness, respect, and continuity during communication (3–6). The improvement in trust observed here may therefore reflect more than increased risk information. It may result from the combined effects of explanation, patient feedback, and participation in decisions. The marked improvement in risk understanding is also consistent with current risk-communication research. Prospect theory suggests that risk judgments are influenced by reference points, loss aversion, and subjective probability weighting (18, 19). Research on message framing further shows that no gain or loss frame has a stable advantage across all medical contexts (20–23). We therefore did not use prospect theory to persuade patients to accept treatment. Instead, the intervention established clear references and presented treatment benefits, risks, and consequences of no treatment together. It also used more understandable expressions of low-probability risk to calibrate risk perception. A 2024 systematic review of informed consent for surgery and interventional procedures reached a similar conclusion. Structured risk-communication aids improved patients' understanding and recall of statistical risk (25). These findings have direct implications for hospital communication management. Traditional preoperative communication often ends when risk disclosure and informed consent are completed. However, a physician's disclosure does not necessarily mean that the patient understands. Communication quality should therefore be assessed by more than procedural completion. Evaluation should consider whether patients understand key information, can restate major risks, have opportunities to speak, and receive responses to core concerns. Preoperative communication can then move from an experience-based personal practice to a service process with defined steps and quality feedback.

### 5.3 Treatment acceptance: from information acquisition to decision quality

Treatment acceptance was markedly higher in the intervention group, and the adjusted OR remained 3.491. This finding is consistent with the shared decision-making principle that patients should understand options and participate in value trade-offs (7). Recent reviews show that patient decision aids and shared decision-making improve knowledge and participation while reducing decisional conflict (9, 10). Recent surgical evidence also indicates substantial room to improve shared decision-making. Its quality may also be related to patient-reported outcomes (14–17). The higher treatment acceptance observed here should not be interpreted as successfully persuading more patients to accept treatment. A more appropriate interpretation is that structured communication may help patients reach clear decisions earlier. This may occur after they understand risks, benefits, and their own preferences. The goal of high-quality communication remains an informed choice that is consistent with patient values. It is not simply a higher treatment-acceptance rate.

### 5.4 Thirty-day complications after surgery or invasive procedures: a patient-safety signal requiring cautious interpretation

The intervention group had a markedly lower risk of 30-day complications after surgery or invasive procedures. The unadjusted RR was 0.419, and the adjusted RR from the modified Poisson model was 0.413. The direction remained consistent among patients with recorded procedure type and among those who clearly underwent surgery. Consistency across models and samples suggests that the association was unlikely to result entirely from model choice, missing procedure type, or inclusion of nonsurgical patients. The direction of this finding is consistent with recent research on preoperative patient education (30–34). One possible behavioral mechanism involves better understanding of preoperative preparation, medication adjustment, postoperative activity, wound care, and recognition of abnormal symptoms. Such understanding may improve adherence and timely reporting of problems, thereby supporting perioperative safety. However, the observed effect size was large, so strong causal interpretation should be avoided. The proposed behavioral mechanisms were not directly measured in the present study and should therefore be regarded as plausible explanations rather than demonstrated mediating pathways. In addition, because the intervention and control groups were drawn from different hospital campuses, unmeasured campus-level differences in physician teams, clinical pathways, anesthesia practice, or perioperative management may also have contributed to the observed difference in complications. Future hospital evaluations could monitor preoperative preparation, patient adherence, recognition of abnormal symptoms, unplanned revisits, and complications together. This would help clarify how risk communication may influence patient safety.

### 5.5 Medical disputes: exploratory findings and the governance limits of communication interventions

Unlike risk understanding, trust in physicians, and treatment acceptance, medical disputes within 3 months did not differ significantly between groups. The adjusted OR was 0.596, but the 95% CI was wide and crossed 1. Only 16 medical disputes occurred in the full sample. Current evidence is therefore insufficient to show that structured communication reduces the risk of medical disputes. Disputes are also influenced by care quality, financial burden, outcome expectations, complaint handling, and mediation mechanisms. Patient-centered communication may improve physician-patient interaction by increasing trust and reducing misunderstanding (1). However, better risk understanding and trust cannot be assumed to reduce disputes directly. The supported conclusion should therefore remain limited. Structured communication improved the cognitive, relational, and decision outcomes that it directly targeted. Medical disputes showed only a directional difference and require larger samples and longer follow-up. In practice, communication interventions should form part of comprehensive hospital risk governance. They should be combined with quality control, complaint management, dispute mediation, and organizational support. They should not be treated as a stand-alone strategy for dispute prevention.

### 5.6 Theoretical contributions

The theoretical contribution of this study is not simply the simultaneous use of prospect theory and the ultimatum game. It clarifies the distinct functions of the two theories in physician-patient communication. Prospect theory explains how reference points, loss aversion, and probability weighting can produce biased risk perception. The ultimatum game explains why a medically reasonable proposal may still be rejected when patient participation and procedural fairness are inadequate. Based on this division, the study translates abstract theory into two operational intervention pathways. The first targets risk-information processing through dual reference points, balanced benefit-risk presentation, and calibration of low-probability risks. The second targets interaction through proposal, patient feedback, physician response or revision, and shared decision-making. These pathways correspond to risk understanding and trust in physicians, respectively, and both relate to treatment acceptance. Together, they form a theoretical chain from cognitive quality to relational quality and then decision quality. Future studies should measure these psychological and interactional variables directly. Prespecified mediation models should test whether cognitive debiasing and interactional fairness actually transmit intervention effects to behavioral outcomes.

### 5.7 Management implications and practical recommendations

The management value of this study lies in reframing physician-patient communication as a service process. It can be standardized, evaluated, and embedded in healthcare quality management rather than relying mainly on individual physician experience. Better risk understanding and trust indicate that communication quality should not be judged only by completion of informed disclosure. Hospitals should also assess whether patients truly understand key information, have adequate opportunities to speak, and receive responses to major concerns. Higher treatment acceptance suggests that structured communication may reduce repeated explanations and decision hesitation caused by insufficient information, risk misjudgment, or low trust. This could improve coordination between preoperative preparation and subsequent care. The lower rate of 30-day complications after surgery or invasive procedures also suggests a possible link between communication management and patient-safety management. Risk communication may therefore become a potential upstream component of perioperative quality improvement.

These findings suggest three practical directions for hospital management. First, hospitals can establish standardized preoperative communication with balanced benefit-risk explanation, patient feedback, and shared decision-making as core steps. This would reduce arbitrary variation in communication across physicians. Second, communication evaluation can move from simple completion rates to outcomes such as understanding, trust, and decision quality. Hospitals could also explore joint monitoring with treatment adherence, recognition of abnormal symptoms, and patient-safety outcomes. Third, moderate stratification can be applied within a common core process. Information format and confirmation frequency can be adapted for older patients, patients with comprehension difficulties, or clinically complex cases. The study did not show a significant reduction in medical disputes. Communication management therefore cannot replace quality control, complaint handling, or dispute mediation. Hospitals still need an integrated risk-governance system combining communication management, patient safety, dispute resolution, and organizational support.

### 5.8 Limitations and future research

This study has several limitations. First, it was conducted at a single tertiary hospital and included patients undergoing elective surgery or various invasive procedures. Generalizability should therefore be tested across regions, hospital levels, and clinical settings. In addition, only two hospital campuses were included, with one campus assigned to each study condition. Consequently, study condition was aligned with campus, and unmeasured campus-level differences could not be statistically separated from the study condition. Patient-level multivariable adjustment reduces confounding from measured individual characteristics but does not fully address this campus-level limitation. Future studies should include a larger number of independently randomized campuses or clinical units. Second, risk understanding and trust in physicians were measured using study-specific 0–10 rating tools. These tools lack extensive independent psychometric validation, which may affect effect-size estimates. Third, multivariable models adjusted for age, sex, disease severity, comorbidities, payment method, and procedure-related factors. However, education, household income, physician seniority, surgical team, anesthesia quality, and perioperative management were not fully recorded. Residual confounding therefore cannot be excluded. Fourth, only 16 medical disputes occurred. The small number of events limited the precision of model estimates, so this outcome should be considered exploratory.

In addition, medical disputes were defined broadly. The study did not distinguish formal complaints, liability disputes, and other dispute types. It also did not record specific causes, severity, or resolution processes. The particular stages that communication might influence therefore cannot be identified. Subgroup analyses were also exploratory, with limited sample sizes and no correction for multiple comparisons. The age-related interaction requires replication in an independent sample. Communication duration, physician workload, and intervention fidelity were not recorded. The implementation cost and sustainability of the model in high-volume hospitals therefore remain uncertain. Future studies should use multicenter prospective designs and validated measures of risk understanding and trust. They should also collect education, health literacy, physician and team characteristics, communication duration, patient adherence, and standardized medical-dispute outcomes. Such studies could test intervention mechanisms, long-term effects, and feasibility in routine hospital management.

## 6 Conclusion

This study integrated the risk-perception mechanisms of prospect theory with interactional fairness inspired by the ultimatum game. It developed a structured physician-patient communication model combining cognitive debiasing and interactional fairness. The model was significantly associated with better risk understanding, greater trust in physicians, and higher treatment acceptance. It was also associated with a lower risk of 30-day complications after surgery or invasive procedures. These main findings remained generally stable after multivariable adjustment and sensitivity analyses. Medical disputes within 3 months were numerically lower in the intervention group but did not differ significantly. These findings suggest that preoperative communication is not only an individual physician behavior. It also has the potential to be standardized and integrated into healthcare quality and patient-safety management. By standardizing risk communication and strengthening patient feedback and shared decision-making, structured communication may improve patient understanding and decision efficiency. It may also support perioperative safety management. Confirmation in studies with a larger number of independently randomized campuses or clinical units is needed before strong causal conclusions can be drawn.

## Data Availability

The original contributions presented in the study are included in the article/supplementary material, further inquiries can be directed to the corresponding author.
